# Pregnancy complications in last pregnancy and mothers’ long-term cardiovascular mortality: does the relation differ from that of complications in first pregnancy? A population-based study

**DOI:** 10.1186/s12905-023-02503-z

**Published:** 2023-07-04

**Authors:** Abdu Kedir Seid, Nils-Halvdan Morken, Kari Klungsøyr, Liv Grimstvedt Kvalvik, Linn Marie Sorbye, Lars Johan Vatten, Rolv Skjærven

**Affiliations:** 1 Department of Global Public Health and Primary Care, University of Bergen, Alrek helseklynge, blokk D, Årstadveien 17, Bergen, 5009 Norway; 2 Center for Alcohol and Drug Research, Aarhus University, Aarhus, Denmark; 3 Department of Clinical Science, University of Bergen, Bergen, Norway; 4 Department of Obstetrics and Gynecology, Haukeland University Hospital, Bergen, Norway; 5 Division for Mental and Physical Health, Norwegian Institute of Public Health, Bergen, Norway; 6 Norwegian Research Centre for Women’s Health, Oslo University Hospital, Rikshospitalet, Norway; 7 Norwegian University of Science and Technology, Trondheim, Norway

**Keywords:** CVD death, Pregnancy complications, Women’s health, Last pregnancy

## Abstract

**Background:**

Women who experience complications in first pregnancy are at increased risk of cardiovascular disease (CVD) later in life. Little corresponding knowledge is available for complications in later pregnancies. Therefore, we assessed complications (preeclampsia, preterm birth, and offspring small for gestational age) in first and last pregnancies and the risk of long-term maternal CVD death, taking women´s complete reproduction into account.

**Data and methods:**

We linked data from the Medical Birth Registry of Norway to the national Cause of Death Registry. We followed women whose first birth took place during 1967–2013, from the date of their last birth until death, or December 31st 2020, whichever occurred first. We analysed risk of CVD death until 69 years of age according to any complications in last pregnancy. Using Cox regression analysis, we adjusted for maternal age at first birth and level of education.

**Results:**

Women with any complications in their last or first pregnancy were at higher risk of CVD death than mothers with two-lifetime births and no pregnancy complications (reference). For example, the adjusted hazard ratio (aHR) for women with four births and any complications only in the last pregnancy was 2.85 (95% CI, 1.93–4.20). If a complication occurred in the first pregnancy only, the aHR was 1.74 (1.24–2.45). Corresponding hazard ratios for women with two births were 1.82 (CI, 1.59–2.08) and 1.41 (1.26–1.58), respectively.

**Conclusions:**

The risk for CVD death was higher among mothers with complications only in their last pregnancy compared to women with no complications, and also higher compared to mothers with a complication only in their first pregnancy.

## Key message

The risk for CVD death was higher among mothers who had either preeclampsia, preterm birth, and/or offspring small for gestational age in their last pregnancy only compared to mothers with any complications in their first pregnancy only.

Studies that focus their attention on first births fail to take into account the risk for long-term CVD death in women who have complications in later pregnancies.

Women´s total reproduction should be considered in studies of pregnancy complications and long-term cardiovascular mortality.

## Background

Cardiovascular diseases (CVD), particularly ischemic heart disease and stroke, are major contributors to disability and the leading causes of death [1]. High blood pressure, unhealthy diet, tobacco smoking, high fasting blood glucose, and elevated low-density lipoprotein are common risk factors attributed to most CVD deaths [2].

Beyond these factors, there is growing evidence that women with a history of pregnancy complications may be at increased risk of subsequent CVD [3–6], and that the risk appears to be higher for women with complications in more than one pregnancy [7]. Wang et al. [8] found that hypertensive disorders in pregnancy, either gestational hypertension or pre-eclampsia, were positively associated with premature maternal death, especially from CVD. In a systematic review, Grandi et al. [9] showed that women with a history of preeclampsia, gestational hypertension, gestational diabetes mellitus, placental abruption, preterm birth, and stillbirth are at increased risk of subsequent CVD morbidity and mortality.

Women with a history of pregnancy loss have a greater risk of CVD [10] and premature mortality up to three decades later, particularly cardiovascular, cancer, and suicide-related deaths [11]. Systematic review and meta-analyses also reported that the risk of CVD was higher among women with prior pregnancy loss (miscarriage, stillbirth, and induced abortion) [12] and pregnancy loss associated mortality is more than double that of delivery associated mortality [13]. In response to the growing evidence, current CVD prevention guidelines in Europe recommend that a woman’s reproductive history should be included as part of her CVD risk assessment [14].

One limitation of most studies is their preoccupation with complications in the first pregnancy, without considering the importance of taking the complete reproduction into account. These studies fail to assess whether events that may occur in more than one pregnancy may modify the results associated with complications in the first pregnancy (e.g. 15). In this study, our aim was to assess the association of pregnancy complications with later risk of CVD death, using information from the women’s total reproduction. As exposures, we used preeclampsia, preterm delivery, and offspring small for gestational age (SGA), because these make up the vast majority of pregnancy complications, and there is substantial knowledge about their association with maternal risk of CVD later in life.

## Materials and methods

We used individual data based on linking multiple Norwegian registers for this study. Firstly, the Medical Birth Registry of Norway (MBRN) was established in 1967 and is based on mandatory notifications of all births in the country from 16 weeks of gestation [15]. The birth notification form includes demographic information and information about maternal health before and during pregnancy, complications during pregnancy or at birth, as well as birth outcomes, including vital status, anthropometric measurements, and neonatal diagnoses. Second, the Cause of Death Registry records dates and causes of death, collected from death certificates for all deaths in the country [16], as well as deaths of Norwegians abroad. Third, the National Education Database (NED) at Statistics Norway contains information on achieved education for all individuals dating back to 1970. All Norwegian inhabitants 16 years of age or older are included, with annual routine updates. The data are categorised based on the number of completed years of education [17]. These three registers were linked using the unique national identification number assigned to each individual at birth or at entry to Norway as an immigrant.

### Sibling data and exclusion criteria

**Fig. 1:**
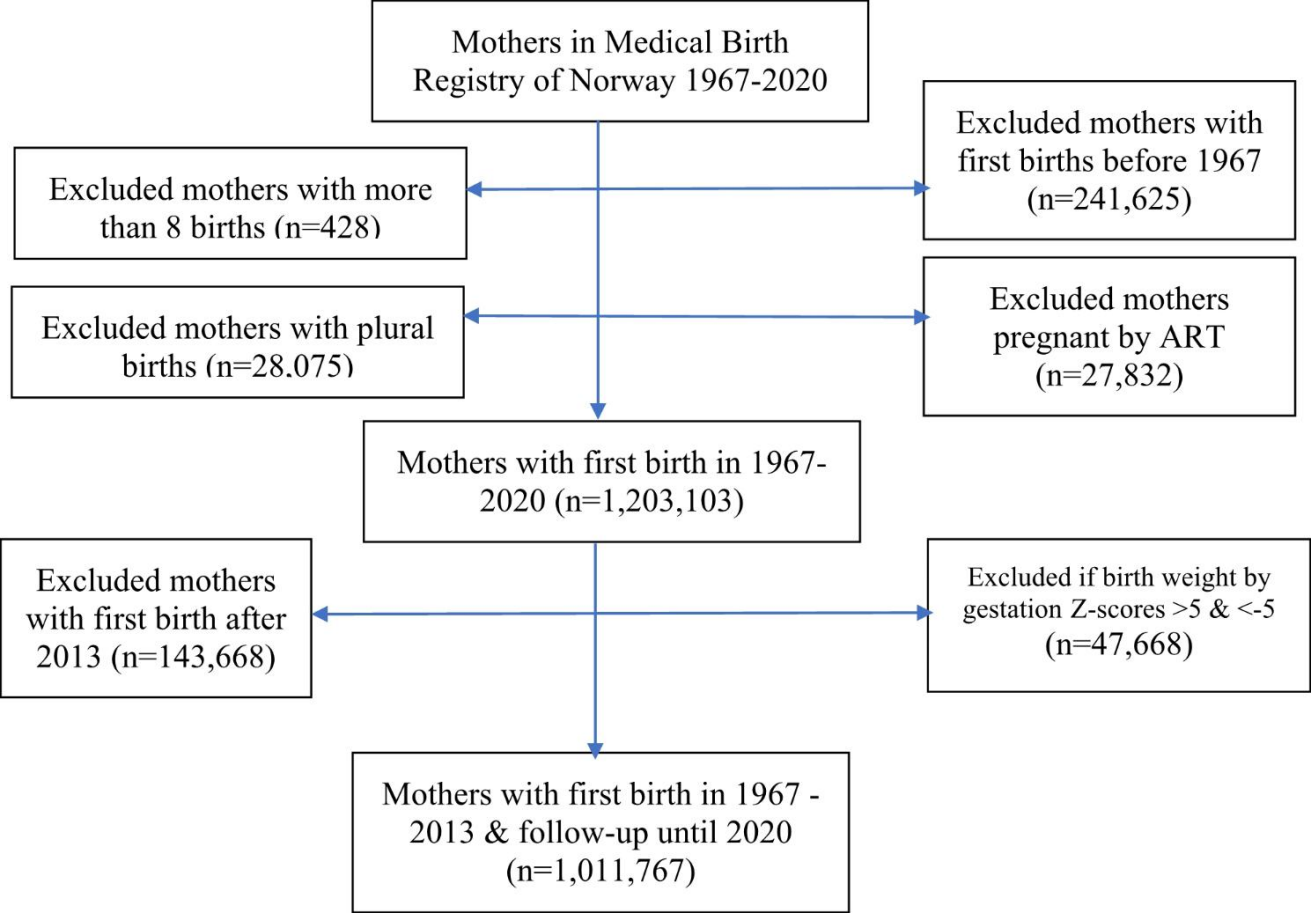
Flow chart of inclusions and exclusions

All births registered in the MBRN during the period 1967–2020 were linked to their mothers, providing sibship data with the mother as the observation unit (n = 1,501,063). We excluded mothers who had their first birth before 1967 (n = 241,625), mothers with more than eight births (n = 428), mothers who were pregnant by assisted reproductive treatment (ART) (n = 27,832), and mothers with plural births (n = 28,075). To give mothers enough time to complete their reproduction prior to mortality follow-up, we excluded mothers who had their first birth after 2013 (n = 143,668). We also excluded mothers where one or more of their infants had z-scores of birth weight by gestational age outside plus or minus 5% (n = 47,668). After these exclusions, the remaining study population for mortality follow-up was 1,011,767 mothers (Fig. 1).

### Outcome variable

Mothers were followed from their last registered birth until CVD death, death from other causes until the age of 70 years, or until the end of follow-up on December 31, 2020, whichever occurred first. The primary outcome was death from CVD before 70 years of age, using the International Classification of Disease (ICD) codes (ICD-8 for 1967–1985; ICD-9 for 1986–1995; and ICD-10 for 1996–2014) to identify CVD deaths as those caused by coronary heart disease (410–414 from ICD-8 and ICD-9; I20-I25 from ICD-10) or cerebrovascular disease (430–438 from ICD-8 and ICD-9; I60-I69 from ICD-10) [18].

### Pregnancy complications

The definition of preeclampsia has been modified over the time period covered by this study. The MBRN has followed guidelines developed by the Norwegian Society of Gynaecology and Obstetrics, and the main criteria, consistent over time, have been an increase in blood pressure to at least 140/90 mm Hg after the 20th week of gestation and proteinuria of ≥ 0.3 g or ≥ 1 + on urinary dipstick [19]. The registration of preeclampsia in the MBRN has been validated and found satisfactory [19, 20]. Preterm delivery was defined as birth prior to the 37th completed gestational week. Offspring SGA was defined as births below the 10th percentile of birth weight by gestational age following Norwegian standards [21]. We defined pregnancy complications as one or more of the complications preeclampsia, preterm delivery, and offspring SGA.

In the analyses, we adjusted for maternal age at first birth as a categorical variable (younger than 20, 20–24, 25–29, 30–34, 35–39, and older than 39 years of age). To account for possible socioeconomic confounders, we adjusted for length of education derived from the National Education Database (low < 11 years, middle 11–14 years, and higher > 14 years). We also stratified the analyses by maternal birth year, grouped as 1922–1948 and 1949–1974. Mothers born after 1974 were excluded from the stratified analyses as they would be too young to study CVD deaths with statistically meaningful numbers.

### Analyses

We reported descriptive statistics as percentages for dichotomous variables and means with standard deviations for continuous variables. Our purpose was to study deaths that occurred until 69 years of age due to CVD, denoted as deaths from CVD. We used Cox proportional hazard models to estimate hazard ratios (HR) with 95% confidence intervals (CI) for the association of pregnancy complications with deaths from CVD. We adjusted for maternal age at first birth and education. Follow-up time was calculated from the time of last birth until CVD death or until censoring or end of follow-up. We adhered to the guidelines for STrengthening the Reporting of OBservational studies in Epidemiology (STROBE). All statistical analyses were performed using Stata 17 software (StataCorp. 2019). The study was conducted in accordance with the Declaration of Helsinki and was approved by the regional ethics committee REK Vest (ref no. 2015/1728, approval date 05.11.2015 and ref no. 2020/13,818, approval date 01.07.2020).

## Results

A total of 1,011,767 mothers who delivered one or more births during 1967–2020 were included in the follow-up, during which a total of 5,062 mothers died from CVD before the age of 70. Table 1 shows characteristics of the study population. Mothers in the second birth cohort (1949–1974) were overrepresented, and most women in that group had at least two births. Most mothers with one or two births had their first delivery between 25 and 29 years of age, whereas mothers with three or more births tended to have their first birth between 20 and 24 years of age.

**Table 1:** Characteristics of mothers (n = 1,011,767) who delivered their first birth from 1967 to 2013 and were followed for further pregnancies and/or CVD deaths until end of 2020

Characteristic	Number of total births			CVD deaths ^a^
	1 birth: N (%)	2 births: N (%)	3 + births: N (%)	N (%)
Mothers’ birth year ^b^				
1922–1948	25 969 (20.0)	63 786 (17.5)	40 246 (14.9)	1935 (38.6)
1949–1974	104 023 (80.0)	301 349 (82.5)	229 753 (85.1)	3080 (61.4)
Total	129 992 (100.0)	365 135 (100.0)	269 999 (100.0)	5015 (100.0)
Mother’s age at first birth (years)				
< 20	10 838 (6.4)	33 765 (6.9)	45 919 (13.2)	753 (14.9)
20–24	44 210 (25.9)	168 674 (34.3)	158 536 (45.4)	2354 (46.5)
25–29	50 747 (29.8)	184 046 (37.4)	111 995 (32.1)	1,267 (25.0)
30–34	37 719 (22.1)	86 111 (17.5)	29 682 (8.5)	481 (9.5)
35–39	20 569 (12.1)	18 186 (3.7)	3060 (0.9)	157 (3.1)
40+	6359 (3.7)	1262 (0.2)	91 (0.03)	50 (1.0)
Total	170 442 (100.0)	492 041 (100.0)	349 283 (100.0)	5062 (100.0)
Mother’s education ^c^				
Low (< 11 years)	38 077 (23.0)	83 973 (17.2)	71 550 (20.6)	2052 (40.8)
Middle (11–14 years)	65 165 (39.4)	194 720 (39.8)	129 546 (37.3)	2265 (45.0)
High (> 14 years)	62 011 (37.5)	210 484 (43.0)	146 313 (42.1)	714 (14.2)
Total	165 253 (100.0)	489 177 (100.0)	347 409 (100.0)	5031 (100.0)

Note: ^a^ Age at death up to 69 years, ^b^ Birth year before 1922 (n = 19) due to small cell in CVD death and birth year after 1974 (n = 246,622) analyses as mothers would be too young to study CVD death with meaningful numbers were excluded in the stratified, ^c^ around 1% (n = 9,928) are missing

**Fig. 2:**
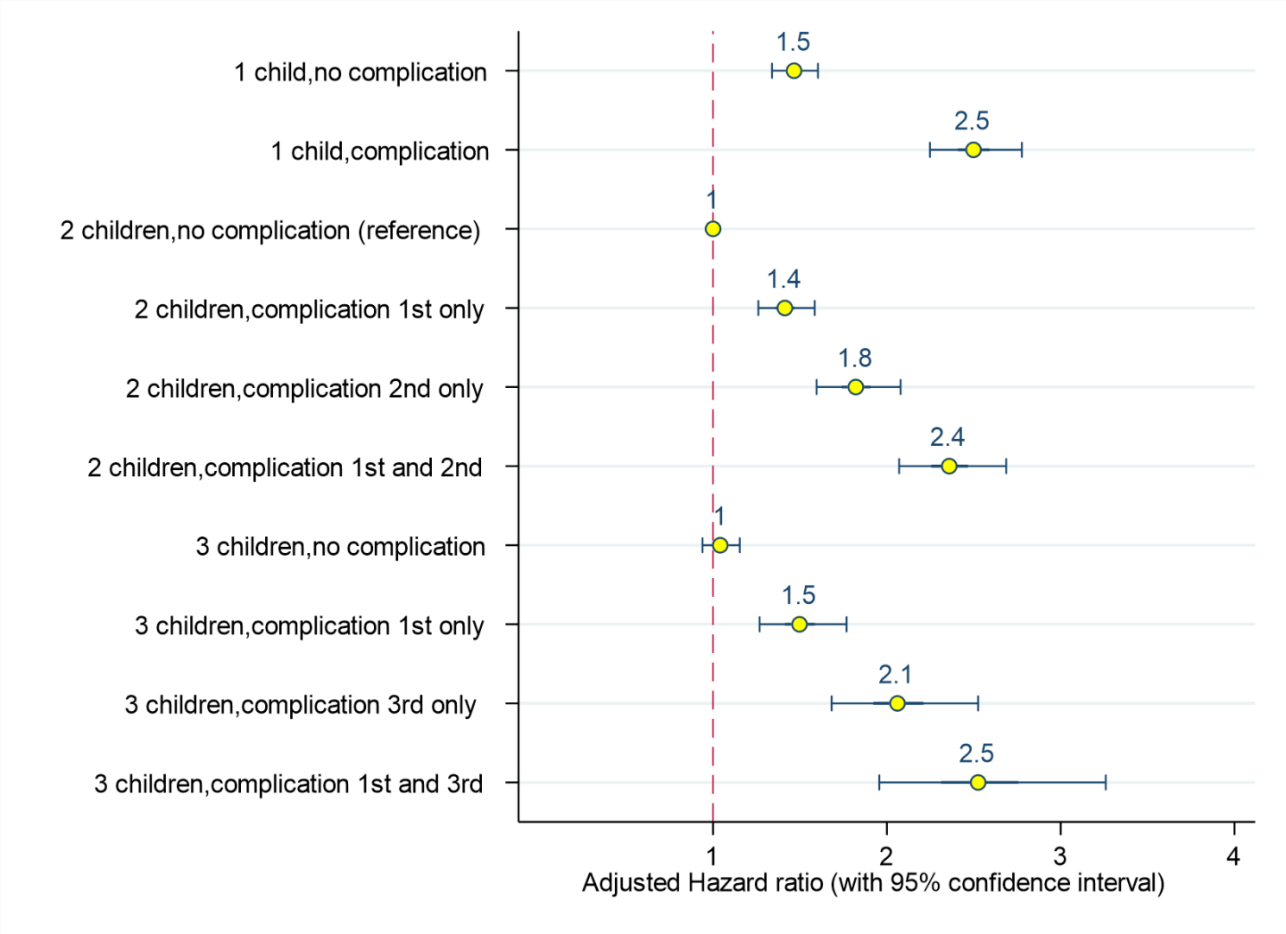
Pregnancy complications (preeclampsia, preterm birth, and/or small for gestational age) by birth order and relation to CVD death adjusted for maternal education and maternal age at first birth

Figure 2 shows the associations of pregnancy complications with the risk of CVD death adjusted for maternal age at first birth and maternal education. We used mothers with two-lifetime births and no pregnancy complications as a common reference, and displayed the results for mothers with one, two or three lifetime births. The results show that mothers who had a complication only in their last pregnancy were at higher risk compared to mothers in the reference group. Also, across all pregnancies, the risk of CVD death associated with complications occurring in the last pregnancy was higher than the risk associated with complications in the first pregnancy.

Table 2 shows associations of complications in either the first or last pregnancy with risk of death from CVD before the age of 70 years. Compared to the reference group (mothers with two-lifetime births and no complications), the risk was, as expected, substantially higher among mothers with a history of pregnancy complications. However, comparing complications of the first and last pregnancy, the risk increase appeared to be higher associated with complications in the last rather than first pregnancy, and persisted after accounting for number of births. For instance, among mothers with three children and a complication in last pregnancy, the risk of death from CVD was twice the risk of the reference group (aHR 2.06, CI 95%: 1.68–2.52), whereas the corresponding risk increase among mothers with a complication in first pregnancy appeared to be lower (aHR 1.49, CI 95%: 1.27–1.76). For mothers with four-lifetime births, the corresponding hazard ratios were 2.85 (CI 95%: 1.93–4.20) associated with complications in the last pregnancy and 1.74 (CI 95%: 1.24–2.45) associated with complications of the first.

**Table 2:** Pregnancy complications (preeclampsia, preterm birth, and/or small for gestational age) in first or last pregnancy by the mother’s total number of births and the relation to maternal CVD death. First births included up to 2013, mothers followed through 2020 (n = 1,011,767)

Reproduction	Total N	CVD deaths ^a^: N (row %)	Crude HR (95% CI)	Adjusted HR (95%CI) ^b^	Adjusted HR (95%CI) ^c^
**One child only**					
No complications	128 586 (14.0)	815 (0.63)	1.54 (1.42–1.69)	1.47 (1.34–1.61)	1.47 (1.34–1.61)
Complication	41 854 (4.6)	510 (1.22)	2.82 (2.54–3.12)	2.50 (2.25–2.78)	2.48 (2.23–2.76)
**Two births**					
No complications	359 911 (39.2)	1275 (0.35)	Reference	Reference	Reference
Complication only first	69 470 (7.6)	384 (0.55)	1.48 (1.32–1.66)	1.41 (1.26–1.58)	1.40 (1.24–1.57)
Complication only last	35 221 (3.8)	267 (0.76)	1.94 (1.70–2.21)	1.82 (1.59–2.08)	1.76 (1.53–2.01)
**Three births**					
No complications	178 612 (19.5)	518 (0.29)	1.03 (0.93–1.34)	1.04 (0.94–1.15)	1.04 (0.93–1.15)
Complication only first	32 744 (3.6)	157 (0.48)	1.54 (1.31–1.82)	1.49 (1.27–1.76)	1.49 (1.25–1.77)
Complication only last	14 795 (1.6)	103 (0.70)	2.24 (1.83–2.74)	2.06 (1.68–2.52)	2.05 (1.66–2.53)
**Four births**					
No complications	35 035 (3.8)	114 (0.33)	1.29 (1.07–1.57)	1.25 (1.03–1.52)	1.26 (1.04–1.54)
Complication only first	6827 (0.7)	34 (0.50)	1.85 (1.31–2.60)	1.74 (1.24–2.45)	1.74 (1.18–2.57)
Complication only last	3149 (0.3)	26 (0.83)	3.19 (2.16–4.70)	2.85 (1.93–4.20)	3.18 (2.16–4.70)
**Five-eight births**					
No complications	8000 (0.9)	38 (0.47)	2.28 (1.65–3.15)	2.15 (1.56–2.97)	2.17 (1.56–3.03)
Complication only first	1595 (0.2)	12 (0.75)	3.27 (1.85–5.77)	2.88 (1.63–5.08)	3.31 (1.83–5.99)
Complication only last	1795 (0.2)	20 (1.11)	5.19 (3.34–8.08)	4.49 (2.88–6.99)	4.55 (2.68–7.71)
Total number of mothers ^d^	917 594	4273	917 594	908 405	896 463

Note: ^a^ death up to 69 years, ^b^ adjusted for maternal education and maternal age at first birth, ^c^ adjusted for maternal education and maternal age at first birth excluding perinatal death, ^d^ missing values in the complication variables led to some reduction in the sample size

In the last column of Table 2, mothers with a perinatal loss in any of her pregnancies were excluded from the analyses, but the results remained essentially unchanged. Associations with death from CVD stratified by mother’s birth cohort period are shown in Table 3. The associations were similar for both periods but slightly more precise for the last period except for associations of mothers with one-lifetime birth due to more mothers in the last period. For instance, in mothers born in the first period who had two-lifetime births and any complication in the last pregnancy, the aHR was 1.74 (CI 95%: 1.40–2.17) compared to 1.83 (1.55–2.17) for those born in the second period. For mothers with three to eight-lifetime births, the corresponding aHR were 1.85 (1.43–2.41) and 2.62 (2.18–3.14). Since there have been large time trends in the absolute risk of dying from CVD, we performed tests of equality of the survival function between the two birth cohorts using the log-rank test and the results showed that there was a difference between the two groups in the probability of CVD death at any time point (p < 0.001, Chi2[1] = 52.02).

**Table 3:** Pregnancy complications (preeclampsia, preterm birth, and/or small for gestational age) in first or last pregnancy by the mother’s total number of births and the relation to maternal CVD death stratified by mothers’ birth year. First births included up to 2013 and mothers followed through 2020 (n = 1,011,767)

Reproduction	Total N	CVD deaths ^a^ : N (row %)	Crude HR (95% CI)	Adjusted HR (95%CI) ^b^
***Mothers’ birth year 1922–1948: N = 118 107***				
**One child only**				
No complication	18 833 (16.0)	334 (1.77)	1.67 (1.45–1.92)	1.61 (1.40–1.85)
Complication	7136 (6.0)	209 (2.93)	2.84 (2.42–3.35)	2.54 (2.15–2.99)
**Two births**				
No complications	45 062 (38.2)	487 (1.08)	Reference	Reference
Complication only first	9599 (8.1)	147 (1.53)	1.43 (1.19–1.71)	1.37 (1.14–1.65)
Complication only last	4,989 (4.2)	97 (1.94)	1.85 (1.49–2.30)	1.74 (1.40–2.17)
**Three-eight births**				
No complications	24 214 (20.5)	227 (0.94)	0.94 (0.80–1.10)	0.93 (0.80–1.09)
Complication only first	5075 (4.3)	76 (1.50)	1.50 (1.18–1.91)	1.44 (1.13–1.83)
Complication only last	3199 (2.7)	63 (1.97)	2.08 (1.60–2.70)	1.85 (1.43–2.41)
***Mothers’ birth year 1949–1974: N = 577 472***				
**One child only**				
No complication	78 035 (13.5)	474 (0.61)	1.45 (1.29–1.62)	1.45 (1.29–1.63)
Complication	25 986 (4.5)	296 (1.14)	2.69 (2.35–3.08)	2.57 (2.25–2.94)
**Two births**				
No complications	218 727 (37.9)	778 (0.36)	Reference	Reference
Complication only first	42 506 (7.4)	234 (0.55)	1.49 (1.29–1.72)	1.42 (1.23–1.65)
Complication only last	22 697 (3.9)	167 (0.74)	1.95 (1.65–2.30)	1.83 (1.55–2.17)
**Three-eight births**				
No complications	144 384 (25.0)	437 (0.30)	1.20 (1.06–1.35)	1.18 (1.05–1.33)
Complication only first	26 995 (4.7)	127 (0.47)	1.73 (1.43–2.08)	1.64 (1.35–1.97)
Complication only last	18 142 (3.1)	140 (0.77)	2.92 (2.44–3.49)	2.62 (2.18–3.14)

Note: ^a^ death up to 69 years, ^b^ adjusted for maternal education

## Discussion

Using linked registry data, we found that the risk of CVD death was higher among mothers who had any pregnancy complications (preeclampsia, preterm delivery, offspring SGA) only in the last pregnancy compared to mothers with a complication only in the first pregnancy.

The higher risk of death from CVD associated with pregnancy complications is in line with previous cohort studies of similar design both in Norway [22, 23] and elsewhere [24]. Specifically, it is well documented that preeclampsia [24, 25], preterm delivery [26], and SGA offspring [9, 27, 28] signal that maternal cardiovascular health may be at risk.

Most of the literature on this topic has studied women’s first birth and ignored the potential importance of complications that may occur in subsequent pregnancies. Thus, studies have typically focused on a single complication related to the first birth and failed to consider the women’s total reproductive history. However, it is already known that preeclampsia in first and cardiovascular risk is strongly modified by women´s subsequent reproduction [29]. For other complications, the evidence is nearly non-existent, but it seems plausible that complications throughout the reproductive period would be important and should be accounted for. In this study, we considered three frequent complications (i.e., preeclampsia, preterm birth, offspring SGA), and we used information from complete (or close to complete) reproductive history of participating women.

As our results suggest, complications in the last pregnancy seem to indicate that future risk may be even higher than that associated with complications in the first pregnancy.

### Strength and limitation

Important strengths of our study include its population base, the prospective design and the unique population registers that allow individual linkage of relevant information. Thus, the MBRN has complete records of all women who have given birth in the country since 1967. Using linkage to the Cause of Death Registry allowed long-term follow-up for cardiovascular mortality while simultaneously taking women´s total reproduction into account. Also, linkage to the National Education Database allowed us to adjust for one of the most important indicators of socioeconomic status.

Thus, our findings contribute to, and extends, the growing literature related to long-term adverse effects of pregnancy complications on maternal risk of CVD. Most importantly, we demonstrate that complications connected to the last birth may be a stronger predictor of cardiovascular disease than complications related to the first birth, and that information on complications in all pregnancies improve predictions.

There are also some limitations to our study. First, we have considered the complete reproduction of a woman in our analyses; however, we were not able to address fully the issue of pregnancy loss in relation to CVD death. Previous studies reported that pregnancy loss (miscarriage, stillbirth, and abortion) is a risk factor for CVD and early mortality [10, 11]. However, when we analyzed our data restricted to mothers who had no lifetime perinatal loss, the results were similar to the main analysis. We acknowledge, though, that additional research addressing pregnancy loss using complete reproduction is needed to help identify the effect of pregnancy complications on the health and longevity of women. Second, it is possible that some unmeasured confounding factors could have influenced the results. For instance, we did not have access to information on behavioural risk factors for CVD, such as smoking, because that information was only included in the MBRN from 1999. Third, we could not account for parental factors related to the health and environmental circumstances of the mothers. Previous studies in Norway [30, 31] and elsewhere [32] have reported that family factors (shared environment and genes) might influence risk factors for a woman´s own pregnancy complications, and subsequently, for cardiovascular health. Finally, most mothers in our sample are younger than 40 years of age at first childbirth, and therefore we could not assess whether the increased CVD risk appears before, during, or after the perimenopausal ages. By using generational data, future research may expand our understanding of these effects, and particularly, it would be of interest to examine whether parental pregnancy complications might contribute to the offspring´s risk of CVD. It should also be added that our results are derived from a universal and nearly free health care system, that might limit the generalizability of our findings to countries with different health care systems.

In conclusion, our results emphasise the importance of using complete reproductive history in studies of pregnancy complications and long-term maternal risk of death from cardiovascular disease. Our findings also suggest that regular follow-up of mothers who have a history of pregnancy complications, especially in their last pregnancy, could be a useful approach to prevent long-term premature cardiovascular death in these women.

## Data Availability

The datasets used during the current study are not publicly available due to privacy restrictions, and so are not available from the authors due to national regulations. However, researchers can apply for similar data by contacting Statistics Norway and the Norwegian Institute of Public Health directly (https://www.fhi.no/en/more/access-to-data/applying-for-access-to-data/).
